# Advances in the Diagnosis of Endemic Treponematoses: Yaws, Bejel, and Pinta

**DOI:** 10.1371/journal.pntd.0002283

**Published:** 2013-10-24

**Authors:** Oriol Mitjà, David Šmajs, Quique Bassat

**Affiliations:** 1 Barcelona Centre for International Health Research, Hospital Clínic, University of Barcelona, Barcelona, Spain; 2 Lihir Medical Centre-InternationalSOS, Lihir Island, Papua New Guinea; 3 Department of Biology, Faculty of Medicine, Masaryk University, Brno, Czech Republic; University of Tennessee, United States of America

## Abstract

Improved understanding of the differential diagnosis of endemic treponematoses is needed to inform clinical practice and to ensure the best outcome for a new global initiative for the eradication of yaws, bejel, and pinta. Traditionally, the human treponematoses have been differentiated based upon their clinical manifestations and epidemiologic characteristics because the etiologic agents are indistinguishable in the laboratory. Serological tests are still considered standard laboratory methods for the diagnosis of endemic treponematoses and new rapid point-of-care treponemal tests have become available which are extremely useful in low-resource settings. In the past ten years, there has been an increasing effort to apply polymerase chain reaction to treponematoses and whole genome fingerprinting techniques have identified genetic signatures that can differentiate the existing treponemal strains; however, definitive diagnosis is also hampered by widespread unavailability of molecular diagnostics. We review the dilemmas in the diagnosis of endemic treponematoses, and advances in the discovery of new diagnostic tools.

## Methods

References for this review were identified through searches of PubMed and WHO databases from January 1, 1905 to January 1, 2013, by use of terms “yaws,” “pian,” “bejel,” “pinta,” “carate,” “endemic Treponematoses,” and “*Treponema pallidum*.” Many articles were identified through searches in the authors' personal files. Articles resulting from these searches and relevant references cited in those articles were reviewed. Articles published in English, French, Spanish, and Portuguese were included.

## Introduction

Treponematoses are infections caused by the spirochetal organisms of the *Treponema* species. These bacteria are the cause of both syphilis (*Treponema pallidum* ssp. *pallidum*) and the so-called nonvenereal or endemic treponematoses (ETs) consisting of yaws (*T. pallidum* spp. *pertenue*), bejel (or endemic syphilis) (*T. pallidum* spp. *endemicum*), and pinta (*T. carateum*) [1]. Dilemmas exist in the diagnosis of patients with ETs because clinical findings do not always accurately identify patients with the disease, and serologic methods are unable to differentiate these disease entities from venereal syphilis and from each other [2]. Furthermore, in resource-poor countries with high rates of syphilis and poor laboratory diagnostics, establishment of the diagnosis of yaws, bejel, and pinta can be even more difficult.

The WHO has now embraced yaws and dracunculiasis (Guinea worm disease) as the only two diseases targeted for eradication on its official list of 17 neglected tropical diseases (NTDs) [3]. As progress is made in yaws eradication in many countries, it becomes increasingly important to acquire more information on a number of aspects of the disease in order to ensure the effective and economical conduct of eradication campaigns. One such aspect is the accurate differential diagnosis with certain skin and other lesions not caused by ETs.

In this review, we focus on dilemmas in the diagnosis of endemic treponematoses in children and adults. We review the clinical presentation and differential diagnosis of the disease, use of epidemiological data, and interpretation of serological tests results, and draw attention to advances in specific diagnostic tools and the use of molecular biology to increase the sensitivity and differentiate the existing strains of pathogenic treponemes [4], [5].

## Epidemiological and Geographical Differences

For differentiation of treponematoses, a point that might give some assistance would be residence in an area where one of these treponematoses was preponderant or present to the exclusion of the others. Transmission of ETs appears to be defined by climate and opportunity because *T. pallidum* is readily killed by drying; it can survive only briefly outside the body (one to two hours) [6]. Yaws and pinta are found in warm, moist climates, mainly in forested tropical regions, and are transmitted by direct skin-to-skin contact. Lesions are promoted by high atmospheric humidity, increasing their oozing and infectiousness [7]. Bejel is found in drier climates, but the bejel treponeme takes shelter in moist areas of the body, like the mouth, thus the infection spreads by direct contact (e.g., children kissing their siblings) or indirect contact through infected communal utensils [8], [9].

**Figure 1:**
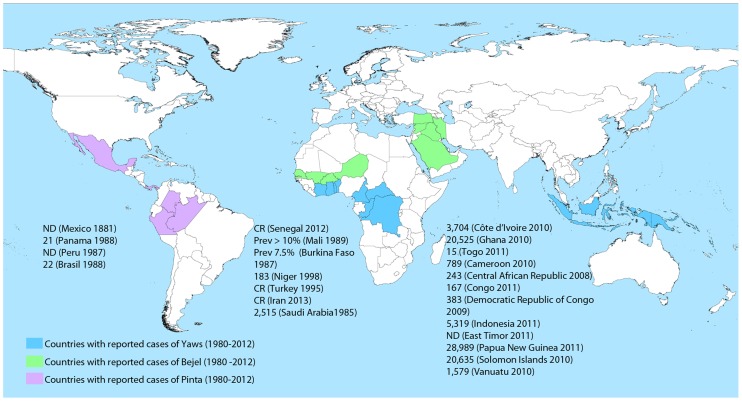
Countries with reported data on yaws, bejel, and pinta from 1980 to 2012 [10]–[29], [32]. Data are number of cases reported in a year, unless otherwise indicated. Prev: prevalence. CR: Case report. ND: No data. Shading indicates the countries where cases of endemic treponematoses have been reported within the last 30 years. In some countries the transmission may be restricted to small areas, rather than affecting the entire country. India interrupted transmission in 2004 and declared elimination in 2006. Since 2004, no new cases have been reported. The principal sources of data are WHO reports [10], [16], [24] and scientific articles identified through searches of PubMed about epidemiology of yaws [11]–[15], bejel [17]–[23], and pinta [25]–[27], [29].

The upsurge in ETs observed in the period 1980–2012 (after they were almost eradicated by the mass penicillin treatment campaigns of the 1950s and 60s) is still poorly understood but is probably related to lack of follow-up care. Their current geographic extent remains uncertain, but there is growing evidence that the number of cases in some countries continues to increase. **Figure 1** shows the countries where cases of yaws, bejel, and pinta have been reported in the last 30 years, though underreporting is likely to be common.

Yaws is the most prevalent of the ETs [10]. Fourteen countries have reported cases of yaws to the WHO in the past three years, including Ghana, Papua New Guinea, and the Solomon Islands, which each reported over 20,000 cases in 2010 or 2011 [11]–[15]. Bejel is encountered in the arid areas of the Sahel (southern border of the Sahara desert), including Senegal, Burkina Faso, Mali, and Niger [16]–[19]. It has also been described in the Arabian Peninsula (Saudi Arabia, Iraq, and Syria) [20], [21]. There was a report of three cases of bejel in southeast Turkey in 1995 (where the disease was considered to be eliminated) [22] as well as a case report from southwest Iran in 2012 [23]. Data on the prevalence of pinta are limited, but it might remain endemic in remote regions of Mexico (states of Oaxaca, Guerrero, Michoacan, and Chiapas), where it was common in the 1980s [24], [25]. It has also been reported among Indian tribes in the Amazon region of Brazil, Colombia, and Peru [26]–[28]. A survey in Panama in the 1980s noted evidence of active or inactive pinta among 20% of the population [29].

Imported cases of yaws and bejel have been documented in children in Europe and the United States [30]–[32], and a case of local transmission of bejel was reported in 2012 in Canada among a family who had lived in a refugee camp in the Republic of Senegal and whose children were all infected [33]. Diagnosis of imported cases is hampered by the limited knowledge and lack of awareness among health care workers in western countries, who may need to include them in the differential diagnosis.

## Distinct Clinical Features

The clinical symptoms and signs of ETs have been widely described and illustrated (**Table 1**) [1], [2], [7]. ETs share clinical features with a number of other conditions common in the tropics and serologically negative suspected cases should be investigated for the aetiology of those lesions (**Table 2**) [34], [35]. Broadly speaking, bejel can be considered to be a semimucosal disorder and yaws a cutaneous condition, which are both moderately invasive producing bone and cartilage involvement in the secondary stage; pinta is a noninvasive condition causing only local dermal lesions.

**Table 1:** Characteristics of the four treponemal diseases.

	T. p. pallidum	T. p. pertenue	T. p. endemicum	T. carateum
**Epidemiology**				
**Geographical distribution (climate)**	Worldwide	Tropics (hot, humid areas)	Deserts of Africa and Saudi Arabia (hot, dry areas)	Central and South America (hot, humid areas)
**Age group (peak incidence)**	Adults (18–30)	Children (2–10)	Children (2–10)	Adults (15–50)
**Transmission**	Sexual and congenital	Skin-to-skin contact	Mouth-to-mouth or utensils	Skin-to-skin contact
**Clinical characteristics**				
**Initial lesion (location)**	Common (genitals)	Common (lower extremities)	Rare (oral mucosa)	Common (extremities)
**Dissemination**	Widespread and systemic	Widespread to skin and bone	Limited to intertriginous areas and facial bone	Limited only to skin
**Late complications without treatment (%)**	Gummas (10%), neurological (10%), cardiovascular (10–15%)	Destructive lesions of skin and bones (10%)	Destruction of nose/palate	Local skin hypo- achromia

**Table 2:** Differential diagnosis for the mucocutaneous manifestations of endemic treponematoses.

Yaws	Pinta	Bejel
**Primary and secondary ulcers and papilloma**	**Primary and secondary lesions**	**Mucosal and perioral lesions**
Syphilis	Eczema	Oral herpes simplex
Leishmaniasis	Classic or athrophic lichen planus	Aphtous ulcers
Paracoccidiomysosis	Tinea corporis	Angular cheilitis
Pyoderma	Syphilis	Syphilis
Ecthyma	Tuberculoid leprosy	
Tropical ulcer	Psoriasis	
	Pellagra	
	Yaws	
**Papulosquamous secondary lesions**	**Dyschromic lesions**	**Cutaneous primary and secondary lesions**
Syphilis	Pytiriasis alba	Syphilis
Psoriasis	Vitiligo	Condyloma acuminata
Eczema	Tinea versicolor	Molluscum contagiosum
Arthropod bites	Melasma	Seborrheic dermatitis
Scabies	Leprosy	Psoriasis
Dermatophytosis	Erythema dyschronicum perstans	Dermatophytosis
Tuberculoid leprosy		
**Tertiary gummatous lesions**		**Tertiary gummatous lesions**
Syphilis		Syphilis
Lupus vulgaris		Lupus vulgaris
Deep fundal infections		Deep fundal infections
Mycobacterial infection		Mycobacteris infection
Rhinosporidiosis		Rhinosporidiosis
Rhinoscleroma		Rhinoscleroma

**Figure 2:**
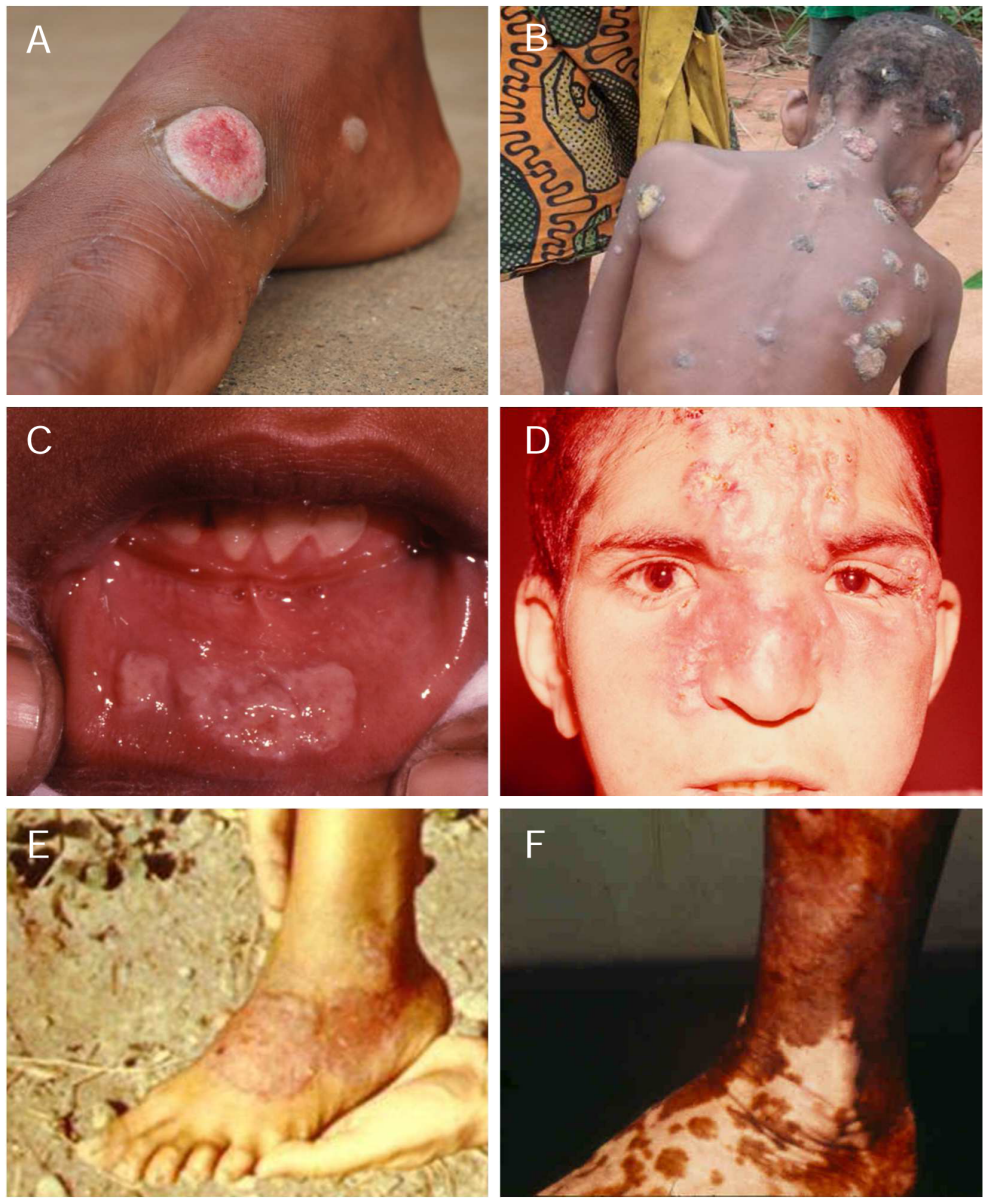
Common yaws, bejel, and pinta lesions in 2013. Papillomatous primary yaws lesion (A); disseminated papilloma of secondary yaws (B); labial mucosal plaques of primary bejel (C); disfiguring infiltration of the nose, glabella, and forehead in a patient with secondary bejel (D); squamous plaque of primary pinta (E); late achromic pinta (F). Sources of photographs: O. Mitjà, Papua New Guinea (A, B); A. Abdolrasouli, Iran (C, D); F. Gómez, Mexico (E, F). The individuals photographed for this publication were informed of the purpose of the photograph and they agreed to have their photograph taken and potentially published.

The clinical manifestations of ETs occur in three distinct stages (**Figure 2**). In yaws, the primary lesion—the so-called mother yaw—is usually a localized papilloma or solitary ulcer 2–5 cm in diameter that may be mistaken for cutaneous leishmaniasis, tropical ulcer, or pyoderma [35]. Yaws skin ulcers are typically circular in shape, have central granulating tissue and elevated edges. Tropical ulcers, caused by anaerobic fusobacteria and *Treponema vincentii*, are painful and characteristically produce a disagreeable odour and blood-stained discharge, which are rarely met in yaws. *H. ducreyi* may be an emerging aetiological agent in chronic extragenital skin ulcers in patients from the Pacific Island region [36]. In contrast, the pinta primary lesion is an itchy, scaly papule or plaque that expands to greater than 10 cm but does not ulcerate [37]. In bejel, the primary lesion is seldom seen because of its small size and location within the oral and oropharyngeal mucosa [37]. Primary lesions in yaws and pinta are most commonly found on the exposed lower extremities, on the legs and ankles (65–85% of cases) [35], [38] but also on the buttocks, arms, hands, and face.

Secondary skin lesions are very different in each of the ETs. Yaws is the most polymorphous condition, mainly affecting the tegumentum with papulosquamous lesions that must be distinguished from common disorders such as psoriasis, dermatophytosis, or crusted scabies [34], [35]. Secondary yaws lesions usually heal spontaneously after three to six months, though infectious relapses may occur for up to five years. The most common secondary-stage manifestations of bejel, according to the analysis of 3,507 cases in Iraq, include mucosal eroding lesions and plaques in the oropharynx and the lips, which can extend locally to the larynx causing hoarseness [39]. Syphilis should always be considered in the differential diagnosis of bejel; both bejel and syphilis may present with mucosal lesions, papules at the oral commissures, and intertriginous condyloma lata [40].

In pinta, lesions remain active and infectious for many years [41] leading to extensive depigmentation and acral dyschromia. Disorders associated with dyspigmentation, such as tinea versicolor, vitiligo, melasma, and leprosy, are often in the differential diagnosis of pinta [42], [43].

Osteoperiostitis is present in yaws, bejel, and syphilis; progressing slowly; it presents as diffuse cortical thickening followed by bone deformation. This mainly affects the long bones (i.e., forearm, tibia, or fibula) and it is generally bilateral in ETs and unilateral in syphilis. Dactylitis is more common with yaws and it may be difficult to distinguish from sickle-cell anaemia [44]. Gummatous lesions, which may be seen in tertiary yaws and bejel, may resemble manifestations of cutaneous tuberculosis, leishmaniasis, deep fungal infection, or mycobacterial infection. Gumma are neither clinically nor histologically specific; the presence of plasmocytes is nonetheless evocative [45], [46]. A dramatic reduction in the number of late-stage cases with destructive lesions has been noted in recent years [47].This is likely the consequence of more accessible health systems, resulting in earlier diagnosis, and the widespread use of antibiotics [47].

Patients with ETs generally do not present with organ involvement, whereas syphilis preferentially affects the central nervous and cardiovascular systems [48]. Neurologic and ophthalmologic abnormalities possibly caused by yaws and bejel have been reported, but without firm evidence of a causal relationship [49]–[51]. Also, it has been suggested that tertiary yaws can cause cardiovascular disease on basis of a large analysis of autopsies in Ghana [52].

## Direct Diagnostic Methods

Direct diagnostic methods are limited by the fact that the *T. pallidum* treponemes cannot be cultured on synthetic media. The rabbit infectivity test (RIT) is the gold standard for demonstrating *T. pallidum* infection, but is impractical for clinical use because of high costs and delayed test results. If direct methods are pursued, treponemes may be identified in a wet preparation of material obtained from early lesions by dark-field microscopy. Direct fluorescent antibody tests using anti–*T. pallidum* antibodies can distinguish pathogenic treponemal infections from saprophyte treponemes [53], [54]. Microscopy, however, is impractical in the field and its sensitivity may be severely decreased if the bacterial load is low or viability of the treponemes is reduced by oral antibiotics or topical antiseptics [55].

The skin pathology using the silver impregnation technique in ETs is largely similar to that of venereal syphilis and despite the existence of relative differences, these cannot be used to differentiate them. The early lesions of yaws show epidermal hyperplasia with collections of neutrophils, and a typical plasmocytic dermal infiltrate [56], [57]. The histopathologic picture of bejel closely resembles that of yaws; though in early bejel, granulomas consisting of epithelioid cells and multinuclear giant cells may be present [58]. In pinta, there is no ulcer formation comparable to that in yaws [59]. In the early lesion, there is loss of melanin in basal cells and liquefaction degeneration. Epidermal atrophy and the presence of many melanophages in the dermis are typical findings of late-stage pinta [60].

## Serological Tests

The serological tests used to diagnose ETs are the same as those used to diagnose syphilis [61]. Serologic testing traditionally involves a nonspecific nontreponemal antibody test followed by a more specific treponemal test for diagnostic confirmation [62]. Nontreponemal agglutination tests, RPR or VDRL, are positive in untreated cases; treponemal tests TPHA, TPPA, and FTA-Abs are more specific, but remain positive for life [63], [64]. The equipment and personnel requirements, such as refrigeration for storing reagents, centrifugation for separating serum, an electric rotator for operating flocculation tests, and experienced laboratory staff for conducting and interpreting the tests, are often barriers to conducting these laboratory-based tests in low-resource settings in developing countries or during yaws community screening campaigns.

Rapid point-of-care (PoC) treponemal tests have become available in the form of immunochromatographic strips; these can be used with whole blood and do not require refrigeration [65]–[67]. However, the treponemal rapid test is not able to differentiate between active and treated infection and unfortunately its result correlates poorly with that of active infection, especially in areas where prevalence of yaws is low or after one or more rounds of mass treatment. Therefore, diagnosis based on the result of a treponemal test alone is likely to result in unnecessary treatment and increases probability of selection for antibiotic-resistant pathogens.

A combined point-of-care test which detects both treponemal and nontreponemal antibodies has been evaluated for the diagnosis of syphilis [68], [69]. The test has been designed for field use, not requiring any kind of laboratory support and it could help to detect yaws cases in isolated rural settings. It could also assist in the surveillance that will be needed for years after endemic communities are treated with antibiotics during eradication programs. In addition, measurement with an automatic reader has the potential to monitor the changes in density of nontreponemal antibodies (corresponding to the titer on nontreponemal test) and allow for quantitative serological follow-up. However, as of this year, there was limited published data in this regard to allow for exploration of their accuracy in syphilis or ETs. More research on these combination tests is highly warranted.

Caution should be exercised in interpretation of the any of the above-mentioned serologic tests results for yaws: i) the antibody response to treponemal infections is often not detectable during the first one to three weeks of infection [70], ii) diagnosis of congenital syphilis can be confused by transferred antibodies from the mother, and most importantly iii) serological tests cannot distinguish between endemic treponematoses infections and syphilis [71], [72]. The whole genome analysis of treponemes is an important avenue of current research, in an attempt to identify new targets for strain- and subspecies-specific molecular and serological diagnosis.

## Whole Genome Sequencing: New Diagnostic Targets

Historically, syphilis and yaws treponemes were considered to be separate species (based on differences in clinical manifestations of the corresponding diseases), but since 1984 they have been classified as subspecies [73], based on DNA hybridization experiments [74]. The whole genome fingerprinting technique [5], [75] revealed high sequence relatedness among all investigated *Treponema* genomes, with the most divergent genomic sequence found in the rabbit pathogen (*T. paraluiscuniculi*). However, *T. paraluiscuniculi* differed from syphilis-causing strains in less than 2% of the genome sequence [76]. These data indicated that complete, high-quality sequences were required for treponeme genome comparisons. *T. carateum* (pinta agent), unlike *T. p. pertenue* or *T. p. endemicum*, is not currently classified as a *T. pallidum* subspecies since our molecular knowledge at this time is insufficient to warrant such a classification.

The whole genome analyses of a number of pathogenic treponemes have been performed, including five *T. p. pallidum* strains (Nichols, SS14, Chicago, DAL-1, and Mexico A) [77]–[80], three *T. p. pertenue* strains (Samoa D, CDC-2, and Gauthier) [81], and other genomes have been sequenced but not published yet (the Fribourg-Blanc simian isolate, *T. p. endemicum* strain Bosnia A) [82]. Genome size, percentage of sequence identity with *T. p. pallidum*, and selected diverse genetic regions are shown in **Table 3**. In all strains, no major rearrangements and a nearly identical gene order was found, further establishing the close genetic relationship between these *Treponema* pathogens. The observed differences, in the presence of restriction target sites, grouped *T. p. pallidum* strains into a separate cluster compared to *T. p. pertenue* strains. Analyses revealed a closer relationship between the Fribourg-Blanc simian treponemes and *T. p. pertenue* strains, and showed that *T. p. endemicum* (Bosnia A strain) also clustered with *T. p. pertenue* strains, though more distantly than that of the Fribourg-Blanc isolate. The genomic data have provided new opportunities to study pathogenic treponemes and have revealed chromosomal regions with accumulated genetic diversity between the subspecies [82].

**Table 3:** Genomic features and historic overview of selected genetic differences found in nonvenereal treponemes.

	T. p. pallidum	T. p. pertenue	Fribourg-Blanc	T. p. endemicum^a^	Year (Ref.)	
**Genome features**						
Genome size (kbp)	1138.0–1140.0	1139.3–1139.7	1140.5	1137.7		
Genome sequence identity with sequences of T. p. pallidum (%)	N/A^b^	99.8	99.8	∼99.7	1998 [77], 2008 [78], 2012 [79]–[81], [96]	
GenBank acc. no.	AE000520.1	CP002374.1	CP003902.1	N/A		
	CP000805.1	CP002375.1				
	CP001752.1	CP002376.1				
	CP003115.1					
	CP003064.1					
**Selected genetic differences** c						
**Differential genetic locus** (type of change)	**Position in gene/genome** (according to CP002374):					
***tpf1***, TP1038 (nt change)	122^d^/1136540	A	G	G	G	1989 [91]
***tpp15***, 5′-flanking region, TP0170 (nt change/Eco47III digestion pattern)	759/191605	C/cut	T/no cut	T/no cut	T/no cut	1998 [92]
***gpd***, TP0257 (nt change/PleI digestion pattern)	579/269535	A/no cut	G/cut	G/cut	G/cut	1999 [93]
***tprC***, TP0117(nt change/BsrDI digestion pattern)	1726–1733/134662–134669	CATTG/cut^e^	TATTA/no cut	CATTG/cut	CATTG/cut	2006 [4]
***tprI***, TP0620 (nt change/BsrDI digestion pattern)	1759–1766/672713–672720	T/no cut	T/no cut	T/no cut	C/cut	2006 [4]
***arp***, TP0433 (no. of repeats)	547–1266/462430–463149	4–16	3–12	15	8, 10^f^	2007, 2008 [94], [95]
**IGR19**, intergenic spacer/hlyB (TP0027)^g^	20–25/34079–34084	9xC, 12xC	CCCTCC	CCCTCC	CCCTCC	2011 [96]

Genome positions are shown in the *T. p. pertenue* Samoa D genomic sequence (GenBank acc. no. CP002374.1).

a For *T. p. endemicum*, the genome size and the genome sequence identity with *T. pallidum* were estimated based on preliminary data.

b N/A, not applicable.

c Genetic differences between *T. p. pallidum* and *T. p. pertenue* were also determined in *tprJ* (TP0621), in *tp92* (TP0326), and in six genomic regions as described by Stamm et al. (1998) [89], Cameron et al. (2000) [90], and Mikalová et al. (2010) [5], respectively.

d Position 123 published by Noordhoek et al. (1989) (*tpf1* vs. *tyf1*); this position is in fact position 122.

e No cut for *T. p. pallidum* Mexico A, SS14, and Sea81-3 strains.

f 8 and 10 repeats found in the *T. p. endemicum* Bosnia A and Iraq B, respectively [95].

g IGR19 is an intergenic spacer between *fliG* (TP0026) and putative hemolysin gene *hlyB* (TP0027) in the Nichols genome (AE000520.1); this region is a part of the *hlyB* gene (TP0027) in the Samoa D genome (CP002374.1).

## Polymerase Chain Reaction Analyses of Clinical Samples

During the past 20 years, there has been an increasing effort to apply polymerase chain reaction (PCR) techniques for direct diagnosis of treponematoses. PCR detection of treponemal DNA is a direct method with detection thesholds as low as a few copies of the treponemal chromosome per PCR reaction (sensitivity of 10^−2^–10^−3^ organism equivalents).

Non-subspecies–specific *T. pallidum* PCR is a developing technology, available as an in-house assay at a few laboratories. Several target sequences have been evaluated with some, such as tpf-1, *bmp*, *tpp47*, *tmpA*, and 47-kDa [83]–[85], encoding subsurface lipoproteins and others, such as *polA* [86], being involved in genome duplication; most recently, *polA* assay has been adapted to real-time TaqMan PCR. [87]. This could be applied to swab specimens for rapid detection of nonvenereal treponemes; however, the low numbers of treponemes in blood put limitations on PCR diagnosis of ETs from blood samples [88].

**Figure 3:**
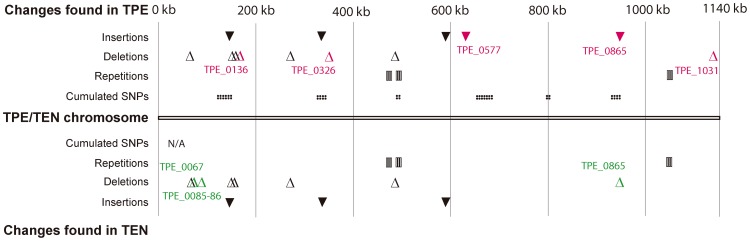
Identified genetic loci showing sequence differences in *T. p. pertenue* and *T. p. endemicum* [**1]**, [81****] **.** These regions are potentially suitable for detection of TPE or TEN strains; however, further testing of these regions in additional strains is needed. Indels specific to tested TPE and TEN strains are shown in red and green, respectively. Only genetic differences consistently present between all completely sequenced TPE and TPA strains are shown. In the case of TEN, only one strain (Bosnia A) has been analyzed on a genome level. Coordinates of insertions in the TPE genomes are taken from the Samoa D genome (GenBank acc. no. CP002374.1): 148701–148781, 331996–332630, 594549–594600, 629981–629992, 945694–945717; coordinates of deletions (CP002374.1): 72694–72695, 150386–150387, 153904–154035, 158201–158202, 279101–279102, 348027–348028, 492496–492501, 1125682–1125683; coordinates of repetitions (CP002374.1): 462430–463149, 498895–499200, 1051990–1052003. Coordinates of cumulated SNPs (defined as ten or more nucleotide changes present in the 100-bp genome window, CP002374.1): 135500–135599, 148700–148799, 153800–153899, 154000–154099, 331200–331399, 331700–332899, 493000–493099, 672000–672199, 672500–673699, 675300–675899, 800500–800599, 936700–936799, 938000–938099. For TEN Bosnia A, only coordinates of insertions, deletions, and repetitions are shown; coordinates of insertions (according to CP002374.1): 148701–148781, 331996–332630, 594549–594600; coordinates of deletions: 72694–72695, 72719–72734, 94986–94998, 150386–150387, 153904–154035, 279101–279102, 492496–492501, 945694–945717; coordinates of repetitions: 462430–463149, 498895–499200,1051990–1052003. N/A, not applicable.

Investigational PCR assays to distinguish nonvenereal *T. pallidum* subspecies have also been evaluated. *T. p. pertenue* and *T. p. endemicum* genetic signatures have been identified (**Table 3**). The regions of sequence divergence could be used for the molecular detection and discrimination of syphilis, yaws, and bejel strains; this option is not possible with currently available diagnostic tests [4], [89]–[96]. Moreover, recent accumulation of genomic data revealed many genetic regions potentially suitable for the detection of *T. p. pertenue* and *T. p. endemicum* strains (**Figure 3**). However, these regions need to be tested in other strains before selecting the most suitable target for a molecular diagnosis of the yaws- or bejel-causing strains.

Experience with DNA sequencing to confirm a clinical suspicion of ETs is limited. A ten-year-old boy with active skin lesions, who migrated from the Republic of the Congo, was confirmed to suffer from yaws [30], and a one-year-old girl with an oral ulcer was diagnosed with bejel during an outbreak of local transmission in Canada [33]. Real-time PCR is useful for identifying the organism and would be a good test to differentiate between the *T. pallidum* subspecies in a single assay, although such techniques are expensive, and unlikely to be available outside reference laboratories [97].

## Laboratory Support and Approach to Evaluation of the Programme to Eradicate Yaws

New WHO yaws eradication strategy and treatment policies for yaws with azithromycin were issued in 2012. The recommended treatment for eradication is one dose of oral azithromycin to be given to entire populations in areas known to harbour yaws. The mass treatment should be followed by immediate mop-ups and resurveys every 6 months to detect and treat remaining cases until zero prevalence is reached. During all stages of the newly embraced yaws eradication programmes, regular surveillance and laboratory support is essential to assess the progress and identify lacunae and gaps. During the mass-treatment phase, active case-finding and treatment campaigns will be organized and diagnosis will be mainly on clinical findings with serological confirmation [62], [65]. During the verification phase after no new active cases have been reported, a combination of treponemal and nontreponemal serological tests will be required for confirmation of all new clinically suspected cases. Interruption of transmission of yaws disease is measured by zero reporting of cases consistently for three consecutive years and verified by no sero-reactors among a randomly selected sample of children one to five years old [10], [98]. If the new rapid dual test accuracy is determined, this could be easier to perform in the field during the verification phase instead of the traditional tests.

## Discussion

The nonvenereal treponematoses continue to be transmitted among rural communities in developing countries and a risk remains for importation of the disease into areas where it is not endemic, and subsequently for local transmission of the etiologic agent. Clinical identification of yaws, bejel, and pinta has important implications regarding diagnostic approach, case management and prevention strategies during implementation of eradication campaigns. Therefore, the clinical assessment skills among health workers and community agents in endemic areas need to be developed through training courses.

In addition, the diagnosis of ETs is difficult and usually requires serological confirmation; a combination of a treponemal and a nontreponemal test is advisable to exclude past-treated infection, but this is challenging in limited-resource settings. Well-designed studies of diagnostic accuracy are needed for the new rapid and PoC combination assay that add potential value to the standard nontreponemal test because it can be easily performed in the field.

In recent years, much effort has been devoted to the development of molecular techniques that enable health care workers to distinguish between the *T. pallidum* subspecies. However, at present the use of clinically useful molecular biological techniques that affect turnaround time, diagnosis accuracy, and patient outcome, and reduce overall costs, is not in sight. Further support is needed to facilitate and expedite the practical application of these scientific discoveries to clinical medicine.

## 

### Learning Points

- The clinical diagnosis for endemic treponematoses may be difficult because these infections produce lesions which can resemble several other diseases in the tropics; thus, support of laboratory techniques and epidemiologic characteristics is necessary.
- The serological tests are the same as those used to diagnose syphilis. The nontreponemal tests are a better indication of active infection and ongoing transmission in an area.
- Available *T. pallidum* PCR assays can be applied to the diagnosis of endemic treponematoses as a direct method with high sensitivity; new molecular diagnostic techniques to differentiate the existing subspecies of pathogenic treponemes are under development.

## 

### Key Articles

1. Hackett CJ (1953) Extent and nature of the yaws problem in Africa. Bull World Health Organ 8: 129–182.
2. Mitjà O, Hays R, Lelngei F, Laban N, Ipai A, et al. (2011) Challenges in recognition and diagnosis of yaws in children in Papua New Guinea. Am J Trop Med Hyg 85: 113–116.
3. Castro AR, Esfandiari J, Kumar S, Ashton M, Kikkert SE, et al. (2010) Novel point-of-care test for simultaneous detection of non-Treponemal and Treponemal antibodies in patients with syphilis. J Clin Microbiol 48: 4615–4619.
4. Čejková D, Zobaníková M, Chen L,Pospíšilová P, Strouhal M, et al. (2012) Whole genome sequences of three *Treponema pallidum* ssp. *pertenue* strains: yaws and syphilis treponemes differ in less than 0.2% of the genome sequence. PLoS Negl Trop Dis 6: e1471. doi:10.1371/journal.pntd.0001471.
