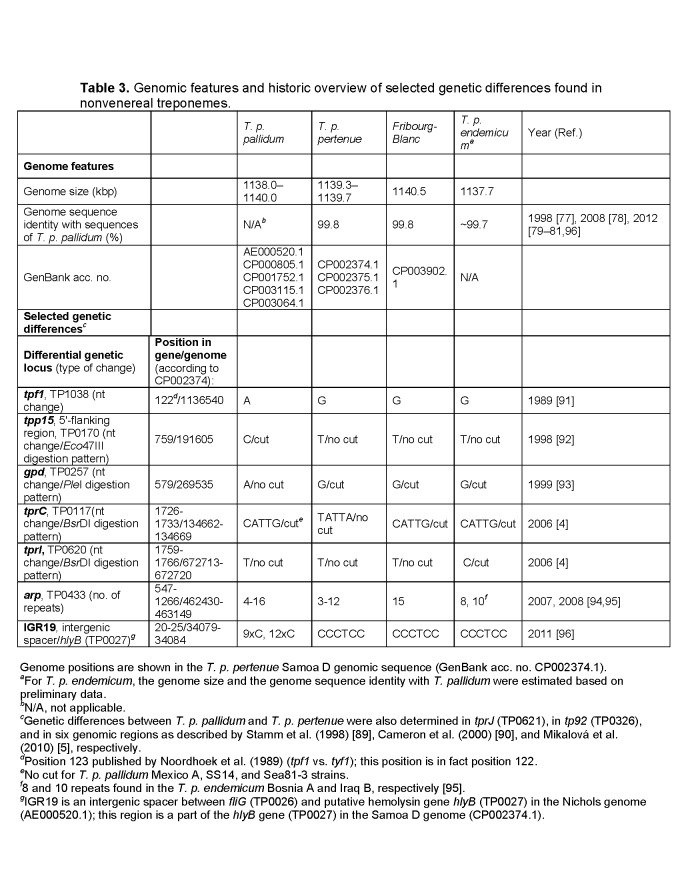# Correction: Advances in the Diagnosis of Endemic Treponematoses: Yaws, Bejel, and Pinta

**DOI:** 10.1371/annotation/20cc3a69-d7d3-49d2-bea4-1759e95a1e09

**Published:** 2013-11-18

**Authors:** Oriol Mitjà, David Šmajs, Quique Bassat

There is an error in the column alignment of Table 3. The column labels, as well as items listed under the 'Genome Features' section, should be shifted one column right. A link to the corrected table can be found here: 

**Figure:**